# State of the art in human risk assessment of silver compounds in consumer products: a conference report on silver and nanosilver held at the BfR in 2012

**DOI:** 10.1007/s00204-013-1083-8

**Published:** 2013-06-19

**Authors:** Bernd Schäfer, Jochen vom Brocke, Astrid Epp, Mario Götz, Frank Herzberg, Carsten Kneuer, Yasmin Sommer, Jutta Tentschert, Matthias Noll, Isabel Günther, Ursula Banasiak, Gaby-Fleur Böl, Alfonso Lampen, Andreas Luch, Andreas Hensel

**Affiliations:** German Federal Institute for Risk Assessment (BfR), Max-Dohrn-Strasse 8-10, 10589 Berlin, Germany

**Keywords:** Silver, Nanosilver, German Federal Institute for Risk Assessment (BfR), Consumer products, Risk assessment, Human exposure, Analytical methods, Silver resistance mechanisms, Assessment and regulation of nanomaterials, Risk communication

## Abstract

In light of the broad spectrum of products containing nanosilver, the harmfulness of nanosilver to human health and the environment was intensively discussed at a conference held in February 2012 at the BfR. The conference agenda covered the aspects of analytics of nanosilver materials, human exposure and toxicology as well as effects on microorganisms and the environment. The discussion recovered major gaps related to commonly agreed guidelines for sample preparation and central analytical techniques. In particular, the characterization of the nanoparticles in complex matrices was regarded as a challenge which might become a pitfall for further innovation and application. Historical and anecdotal records of colloidal silver have been sometimes taken as empirical proof for the general low toxicity of nanosilver. Yet as reported herein, a growing number of animal studies following modern performance standards of toxicity testing have been carried out recently revealing well-characterized adverse effects on different routes of exposure in addition to argyria. Furthermore, recent approaches in exposure assessment were reported. However, consumer exposure scenarios are only starting to be developed and reliable exposure data are still rare. It was further widely agreed on the workshop that the use of silver may lead to the selection of silver resistant bacteria. With respect to its environmental behavior, it was suggested that nanosilver released to wastewater may have negligible ecotoxicological effects. Finally, the presentations and discussion on risk assessment and regulation of nanosilver applications gave insights into different approaches of risk assessment of nanomaterials to be performed under the various regulatory frameworks.

## Analyzing nanoparticles in complex matrices reveals challenging

The recent recommendation of the European Commission on the definition of nanomaterials is emphasizing the determination of the non-bound particles as a number size distribution from 1 to 100 nm (EU Commission 2011). While this definition is precise from a legal point of view, it causes substantial constraints as there are very few or no easy analytical methods to measure this variable without any conversions. An additional limitation is due to the fact that currently only a few already existing analytical techniques can reach far down to 1 nm as required by the definition. Thus, the detailed analytical characterization of the basic particle inherent properties like size, shape and surface area should stand at the very beginning of any further considerations. A thorough characterization of the nanoparticles especially in complex matrices is still the biggest challenge in this research area and could potentially become a pitfall for further innovation in the field.

### Analytical methods, limitations and repository materials

Douglas Gilliland (Joint Research Center JRC—Institute for Health and Consumer Protection IHCP, Italy) discussed the currently available analytical methods. Electron microscopy (EM) enables visualizing of the nanoparticles itself (Linsinger et al. 2012). Although this technique is usually regarded as the standard method for particle size determination, this is only unrestrictedly true for very ideal samples. If the composition of the analyzed sample becomes more complex, for example, due to the formation of aggregates, the determination of the particle size distribution by EM is rather challenging.

Other commonly used techniques are light scattering methods with dynamic light scattering (DLS) being the principal one. For monodisperse samples, DLS can be used to obtain quite precise size measurements, but for the analysis of complex mixtures of “real-life samples,” this technique has some severe limitations. In particular, in the presence of larger particles, a heavy weighting toward the larger particle sizes occurs.

One scientifically accepted alternative overcoming this difficulty is using a whole suite of appropriate techniques in a tiered order. The JRC/IHCP recommends a multi-technique approach integrating optical methods, direct imaging and precise separation methods. One preferred way of analyzing a composition of nanoparticles is the detachment of size separation from the actual size measurement in order to determine the relative quantities in conjunction with the relative particle size. For example, techniques like asymmetric flow field-flow fractionation (AF^4^) or centrifugal particle sedimentation (CPS) are used to fractionate particles into different portion sizes. The purpose of these comparatively gentle separation techniques is the full recovery of particles in their separated state. At this point, a wide variety of different detectors can be used for a more precise nanoparticle characterization. Examples for such detectors, which can be combined with AF^4^, are the refractive index, DLS and the ultraviolet/visible (UV/Vis) detector. To achieve ultimate sensitivity in case of inorganic nanoparticles such as silver, AF^4^ can be connected online to the inductively coupled plasma mass spectrometry (ICP-MS).

At JRC, the following techniques are used for in-depth analyses of the properties of nanoparticles at the molecular level: X-ray photoelectron spectroscopy (XPS) to characterize the surface chemistry of the nanoparticles; synchrotron radiation circular dichroism (CD) to study the various alterations of the protein corona that would occur during interaction of the particles (Laera et al. 2011) and nuclear magnetic resonance (NMR) spectroscopy to monitor any changes which can occur in the conformation of ligands.

Besides instrumentation, the absolute quantification of nanoparticles, the validation of the analytical method of choice and the calibration of the analytical equipment require the availability of appropriately characterized reference materials. In the absence of certified reference materials, which are so far restricted to gold and silica nanomaterials, the creation of a repository of well-characterized representative nanomaterials is essential for the implementation of any analytical test methods. The nanosilver repository material NM300 K provided by the JRC is well characterized, monodispersed, highly concentrated (10 % w/w) with more than 90 % of the material being below 20 nm in size (maxima 10–15 nm). This has been achieved by applying a mixture of two different surfactants as stabilizing agents, that is, 4 % w/w of each polyoxyethylene glycerol trioleate and Tween 20^®^.

As nanoparticles normally interact very quickly with their surrounding media, thereby altering their physico-chemical properties, measurement of the changes in particle size distribution in different media is essential for the understanding of the toxicological effects that may occur. The following results from studies carried out by the JRC may illustrate such alterations. In the presence of a high ionic content solution, the nanosilver particle dispersion becomes unstable. After 30 min in such an environment, the material reveals hardly detectable, and after 60 min, no nanoparticles could be observed anymore either due to aggregation or complete dissolution in the sample. Conversely, when the nanoparticles are kept in a protein-rich environment, like cell culture media, even after 12 h, there is a significant amount of material left in the mere monodispersed state and only after 48 h most of the material begins to disappear from the nanorange. In addition, silver nanoparticles may also remain relatively stable even after 24 h in water possessing low ionic strength. Increasing the ionic content by using water of ascending degrees of hardness, the dissolution or aggregation level of nanosilver particles is simultaneously rising until particles are no more detectable after 24 h. The addition of humic acid revealed as very powerful stabilizing agent, even when applied in very small quantities such as 10 μg per ml. Under such conditions, 50 % of the originally monodispersed nanoparticles remain preserved over 24 h. Studying the dissolution rate of silver particles stabilized with maltose in relation to particle size, it was found that the particles dissolve only between 5 and 10 % in 24 h, and even after around 7 days in solution only between 20 and 50 % of the silver particles had been dissolved. Although the larger particles had the lowest dissolution rate, it is assumed that this effect is only due to the changed surface-to-volume ratio, because the difference between larger and smaller particles was not nearly as significant as expected.

### Experimental challenges analyzing complex matrices

In his talk, Stefan Weigel (RIKILT, The Dutch Institute for Food Safety, The Netherlands) was focusing on the analysis of silver nanoparticles in complex matrices. Recent exposure assessments are hampered by the limited availability of specific methods to determine size and elemental composition of complex matrices as exemplified by consumer products and food. Consequently, there is hardly any validated data on the presence of silver nanoparticles in such products.

A promising technique might be the single particle inductively coupled plasma–mass spectrometry (sp ICP-MS) as it represents a tool for the determination of both the number and mass concentration of silver nanoparticles. Instrument settings and nanoparticle concentration in the sample will be adjusted in a way that individual nanoparticles are being recorded. While the number of peaks corresponds to the particle number concentration, the intensity of the signal is proportional to the mass of the particles (Pace et al. 2011). Under the assumption of spherical non-porous particles, which are not composed of different materials, it is possible to calculate the original size of the particles. The method has successfully been applied to, for example, environmental, biological and food samples. Hyphenated separation and detection techniques have proven to be suited for the quantitative determination of silver nanoparticles in complex media. In particular, AF^4^ is commonly used for the separation of nanoparticles according to their sizes and can be coupled online to a variety of different detectors such as UV, light scattering and ICP-MS (von der Kammer et al. 2011). Screening methods are a cost-efficient alternative to the aforementioned techniques and recently a biosensor method for the detection of silver nanoparticles has been reported (Rebe et al. 2012). This surface plasmon resonance-based assay uses a human metallothionein as a recognition element on the sensor chip surface.

Characterization of nanoparticles is often carried out in pure suspensions with sizing techniques that are not element specific (e.g., light scattering, particle tracking analysis or EM) and/or with element-specific methods (e.g., AAS, ICP-MS, ICP-OES) that are not size specific (determination of bulk concentration). In application media and even more in vivo, the nanoparticles may change considerably. Agglomeration, aggregation, dissolution and attachment to proteins have been described. Therefore, it is essential to characterize nanoparticles in cell culture media or under in vivo conditions by using size and element-specific methods or a combination of methods to achieve a meaningful answer to the experimental question addressed.

A further specific issue is the existence of different solid forms of silver species that via dissolution can be partly transformed into other species. Thus, elemental silver (Ag^0^) slowly dissolves and depending on the chemical environment, the resulting Ag^+^ ions may form soluble complexes such as AgCl_2_^−^, AgCl_3_^2−^, [Ag(NH_3_)_2_]^+^, covalent adducts with thiol group containing compounds (e.g., glutathione, cysteine, proteins) or insoluble salts (AgCl, Ag_2_S) which may precipitate as particulates (Liu et al. 2010). Furthermore, Ag^+^ can be easily reduced to Ag^0^, for example, by glucose, thus leading to *de novo* formation of Ag^0^ nanoparticles. It therefore becomes crucial to determine the identity of the chemical species in which silver nanoparticles are present in the test tube.

In summary, despite the already-mentioned difficulties, a number of different approaches for the detection and characterization of nanoparticles in complex matrices have yet been developed or are currently under development. However, sample preparation is one critical issue for such samples and the formation of artifacts and matrix effects is a common problem (Dudkiewicz et al. 2011). Furthermore, the validation of methods for the analysis of silver nanoparticle’s size, distribution and overall presence is currently severely hampered by the absence of any certified reference materials, both for pure dispersions as well as in various matrices.

## Consumer exposure scenarios are only starting to be developed

Irrespective of the claimed benefit of nanomaterials, their utilization must be safe. As part of the overall risk assessment exposure characterization comprises the systematic identification of potential exposure scenarios and the estimation of exposure levels via oral, dermal and inhalative routes. Direct exposure to pristine manufactured nanomaterials is most likely for workers in industrial settings when handling nanomaterials without protection measures or by accidental release from enclosed automatic production systems. Consumers are mostly intentionally or unavoidably exposed to nanomaterials when using the increasing variety of products meanwhile containing these materials, such as lotions, antiperspirants, shoe sprays and food package materials. Next to the content, an assessment of the resulting exposure will have to take into account the material humans come into contact with. The four talks in this session covered current results of research on textile functionalization, nanoparticle abrasion from surface coatings and content, release and uptake assessments for silver and nanomaterials from food, dietary supplements and consumer products. For the purpose of this review, their contents have been sorted according to exposure routes.

### Inhalative exposure

Among other applications, silver is used as a biocidal agent in sprays to treat shoes and feet, and to act as antiperspirant which can result in exposure via the respiratory tract. Natalie von Götz (ETH Zürich, Switzerland) reported the levels of 6–9 ppm silver and below the detection limit in the solutions of sprays investigated at the ETH Zürich and EMPA (Lorenz et al. 2011a). Subsequent TEM analysis of the corresponding aerosols indicated no silver particles. It was argued that this finding might be due to the silver having been a mixture of silver salts and nanosilver particles, and would need to be elucidated further. A recent US study for comparison detected up to 56 ng nanosilver per spray action in aerosols (Quadros and Marr 2011). A remarkable finding reported by von Götz was that the investigated pump spray did not generate particles in aerosols measurable with a scanning particle mobility sizer after thermodesorption in 80-cm distance to the spraying nozzle. The emitted droplets dropped down by gravity before they reached the detector. However, the propellant gas driven sprays produced many inhalable nanoparticles or nanoscaled droplets irrespective of the original nanoparticle content in the dispersion.

In tiered release experiments, Michael Stintz and co-workers (Technical University of Dresden, Germany) applied increasingly stressing surface treatments to coated parquet. In a first tier, abrasers with sand stone wheels achieved a low-velocity scratching treatment used to simulate an abrasion process comparable to walking on the parquet with sandy shoes. Parquet coatings contained zinc oxide in nanoform (clear coat) and/or titanium dioxide as whitening pigment. In this setting, the treated surfaces released negligible amounts of nanoparticles because of the low velocity and the elasticity of the clear coat (Vorbau et al. 2009). In the second tier, a high-speed rotating sander was used. As a major result, it was impossible to distinguish between the generated aerosols from several surface coatings with nanoparticles (zinc oxide and iron oxide) and from those without (Göhler et al. 2010). In a third step, furniture, parquet and architectural coatings were artificially aged by UV radiation in a dry atmosphere (2,000 h at 50 °C; European Standard for indoor aging) prior to a fully encapsulated sanding procedure. In essence, no free nanoparticle additives (e.g., ZnO) were observed by applying extensive analytical procedures (scanning/transmission electron microscopy and energy disperse X-ray spectroscopy, SEM, TEM, EDX), despite considerable sanding-induced release of particles in the nanoscale. The TEM images revealed that the nanoparticle additives remained firmly embedded in the surrounding matrix material of larger particles, hence the nanoscaled particles observed originated solely from the pure matrix material. Irrespective of the kind of surface coating, additional nanoparticle additives did not have any impact on particle size distribution patterns. However, the patterns and generated amounts were significantly altered by aging. In these cases, more particles were detected and a broader particle size distribution was noted. Stintz estimated that up to 150.000 respirable particles per cm^3^ can be produced through sanding procedures. Reasoning that comparably high particle concentrations are reached in heavy traffic roads during rush hour, he considered it remarkable that special breath masks are recommended especially for long-time exposure of professional sanding workers (Hillemann et al. 2006). Besides standardized procedures for the mechanical treatment of surfaces exist, the workshop did not address a harmonized strategy to determine number concentrations at the workplace (Kuhlbusch et al. 2011).

### Dermal exposure

The intact skin has been shown to be an effective barrier for nanoparticles. For silver nanoparticles, skin penetration rates less than 0.01 % have been published (Larese et al. 2009). Despite such low rates, the internal exposure resulting from high external burden may still be considerable. Lorenz and co-workers have recently published an assessment of potential exposure via cosmetics and personal care products taking into account the behavior patterns of German consumers (Lorenz et al. 2011b). However, the authors did not include creams in their reflections on silver. Based on a reported common application of 0.8 g each on hands and face trice a day and a content of 0.1 % silver, von Götz calculated an external exposure of 4.500 μg silver/day. While internal exposures can be assessed from external burden on the basis of uptake factors, von Götz emphasized that up to date, irrespective of the route regarded, these factors have been determined in only a few studies, if any. Furthermore, these have usually been conducted with a specific nanoparticle material and intact skin. This should be taken into account when employing the factors as they are prone to be influenced by changes of characteristics, such as size, surface coating and form of nanoparticles. In addition, conditions with reduced skin barrier function may enlarge the taken up fraction of particles.

Nina Keusgen (RWTH Aachen Institute for Interactive Materials Research, Germany) presented the results on nanosilver functionalization of textiles for medical purposes. The investigated functionalization techniques are based on the 2–10 nm nanosilver being alternatively: (1) incorporated into polyvinyl alcohol fibers, (2) deposited on organic or inorganic nanocarriers which are fixed to the textile surface via ionic interaction and/or polymers and (3) incorporated in microgel containers to form nanogels for textile finishing. 0.75 % w/w of 2–8 nm nanosilver bound to polyvinyl alcohol fibers was found to inhibit bacterial growth in the fiber efficiently. Fastness and efficiency of the functionalization have been shown to depend on the employed carriers and binding technology. A polycationic finishing prevented elution best. All developed techniques showed superior performance in comparison to a cotton sample finished with a commercial silver salt-based product.

### Oral exposure

Richard Winterhalter (Bavarian Institute for Health and Food Safety, Germany) elucidated sources of nanoscaled materials in food and food supplements. In essence, nanoscaled silver could occur due to unintended contamination during production processes, migration from food contact materials (FCM) or from deliberate inclusion. In regard to the last two sources, he explained that within the European Union, silver as food additive is only authorized for the specific use to make food surfaces more shiny (sweets), which cannot be achieved by nanosilver. Furthermore, up-to-date employment of nanosilver in FCM and in preparations marketed as food supplements is not assessed in the European Union. However, products containing them can be obtained via the Internet. The increasing application of nanotechnology and synthetic nanomaterials or nanoparticles in the production of food and FCM presents a challenge for food safety authorities. The Bavarian Health and Food Safety Authority and the Fraunhofer Institute for Process Engineering and Packaging have therefore set up a project which aims to measure nanoparticle migration from food packages into food, to characterize nanoparticles in food and food supplements and to develop the required sensitive and specific analytics for these tasks. An asymmetric flow field-flow fractionation (AF^4^) in combination with UV and multi-angle light scattering (MALS) detection has been developed for concentration measurement and characterization of silica and silver nanoparticles. While further method development will be needed for refinement of cross-flow rates, solvents and sample preparation, its first application to colloidal silver preparations measured concentrations generally falling significantly short of labeled contents. Additional information given during the following discussions was that migration experiments for titanium from plastic bottles did not lead to levels in the food stimulants exceeding the reference value. However, reasoning that there are studies revealing globally marketed FCM may release up to a few percent of silver, such results should not be generalized.

Von Goetz et al. (2013) also presented results on silver release from FCM. Using ICP-MS and TEM, her group examined four food containers bought in the United States and Germany claiming to contain silver in their material or rubber sealing. With a detection limit of 0.1 μg/g material, this claim could only be verified for two products (18.7 and 37.1 μg/g plastic). Both were subsequently subjected to release experiments. Employing water, ethanol and acetic acid as food stimulants, silver release was observed for one box. Acetic conditions resulted in the highest yield (30 ng/cm^2^). Remarkably, using MD-ICP-MS, an estimated amount of 10–20 % of the silver detected in the simulant was shown to be particulate. To explore the impact of multiple utilization, the food simulant was removed and the box stored for some days before repeating the measurement. In this experimental setup, the silver release dropped markedly. A worst-case assessment was thus based on the assumption of an initial use of the box for storage of acetic food. An easily consumable amount of 100 ml would result in an acute oral exposure to 4.2 μg of silver. While this seems low in comparison to reported silver concentrations in US drinking water in the 60s and 70s of the last century, von Götz reasoned that a comparison to apparently safe water levels could be foiled by the circumstance that a small fraction of the released silver might consist of nanoparticles.

## The current knowledge on the human health hazard of nanosilver is growing but yet limited

### Challenges and pitfalls

The presentations in this session provided both reviews on the existing database and insights into ongoing research aiming at a better understanding and estimation of the human health hazard potential of nanosilver for regulatory purposes. Particular challenges of nanospecific toxicological testing were also addressed. The subsequent plenary discussion stressed the importance of reliable and comprehensive toxicological information for manufactured nanosilver, which in turn requires standardization and harmonization of test materials and methods.

In terms of testing requirements, Helinor Johnston (Heriot-Watt University Edinburgh, UK) emphasized the necessity for extensive characterization of the nanomaterial in a given test medium, since physiochemical properties that drive toxicity may vary dramatically depending on the test system applied. Likewise, dispersion protocols need to be properly documented and justified. Furthermore, the interaction with biological molecules as well as interference with chemical compounds and reagents of the assay system has to be addressed. Identification of crossover challenges in human and environmental studies may also be helpful (e.g., how to cope with agglomeration of nanoparticles). In this context, cross-species comparisons have also been encouraged (Gaiser et al. 2012). Exposure information is relevant not only for risk assessment, but also for interpreting existing hazard information and for adjusting the dosing regimen when designing toxicological studies. Further investigation on the relationship between exposure to a nanomaterial, its effects and its physico-chemical properties, may provide insight into structure–activity relationships and clues on the most appropriate dose metric prompting to describe toxicity. In consideration of the large variety of different nanoforms of the same chemical identity as well as the limitations in resources and access to sophisticated analytical instrumentation, testing and analysis will necessarily face constraints. These may be overcome by intelligent testing approaches. In vitro testing was identified as one important step in this direction since the results might support to generate a solid database for grouping of nanomaterials according to the toxicological potency and, ultimately, fostering the development of high throughput systems. However, currently used in vitro models or those that are under development need to be validated and their predictive values for in vivo toxicity yet to be determined (Kermanizadeh et al. 2012). It was also emphasized that there is a need to identify those physico-chemical properties which trigger mode of action and specific adverse responses, in order to develop structure–activity relationships that are able to predict nanomaterial toxicity. Until this process becomes viable, the present methodological limitations have to be considered carefully in risk assessment.

### The current toxicological database

Susan Wijnhoven (National Institute for Public Health and the Environment, The Netherlands) presented an overview on the available toxicological data for nanosilver (Wijnhoven et al. 2009). Various knowledge gaps precluding meaningful hazard assessment were identified. One of the most important knowledge gaps—the role of ion release in distribution and toxic responses to nanosilver—was addressed by intravenous injection of nanosilver of different particles sizes into rats. Because of distinct distribution patterns observed for each particle size, it was concluded that the nanoparticles could not have degraded immediately (Lankveld et al. 2010). However, ion release behavior may have varied itself with particle size and available surface. With respect to toxicity studies, the database could be improved in recent years, in particular by a number of repeated dose toxicity studies in rodents investigating different routes of exposure (Ji et al. 2007; Kim et al. 2008, 2010; Sung et al. 2009; 2011). In addition, a recent dermal toxicity study in guinea pigs reported local as well as systemic effects (Korani et al. 2011). The repeated dose studies may give valuable input for risk assessment but require thorough evaluation.

Based on these data, preliminary risk assessments have been published for nanosilver (Christensen et al. 2010; Pronk et al. 2009). Since being afflicted with uncertainty, they do not support waiving of further testing of systemic effects. For a more robust risk assessment, more valid data have to be generated both on the exposure and on hazard. For the latter, toxicokinetic investigation of different types of adequately characterized nanosilver materials was encouraged. Other major gaps identified to be investigated with priority include repeated dose toxicity, potential target organs (liver, lung, immune system), genotoxicity, reproductive toxicity, as well as oral, dermal and inhalative studies relevant and designed according to relevant occupational and consumer settings.

### New toxicological data

Wim de Jong (National Institute for Public Health and the Environment, The Netherlands) presented data from a 17-day toxicokinetic study and a 28-day repeated dose study using nanosilver of different sizes (all from nanoComposix). Nanoparticles were administered to rats by daily intravenous injection in order to analyze the systemic distribution and to identify organs at risk of adverse effects by these nanoparticles. The silver content in blood and organs was measured by inductively coupled plasma mass spectrometry (ICP-MS). Early target organs were liver and spleen, while after 5 days, particles had accumulated most in spleen, and also in liver and lungs. The silver content was highest in spleen, liver and lungs for particles of 80 and 110 nm, while 20-nm particles were most prevalent in kidneys. The low recovery of the latter might be attributable to a higher rate of degradation due to a high surface-to-volume ratio. The concentration of silver in blood fell rapidly within minutes after intravenous administration, reaching a plateau at one-third of the initial concentration. After intravenous treatment for 28 days, relative body weight gains were lower in both sexes of rats in the high dose groups (2 and 6 mg/kg bw). In both particle size ranges of 20 and 100 nm, liver weights remained unaffected while spleen weights in the 6-mg/kg bw dose group were increased. Organs as well as lymph nodes showed pigmentation in treated groups. The overall spleen, B and T cell numbers were significantly increased, while natural killer (NK) cells were only slightly increased. By contrast, spleen NK cell activity was decreased at 2 and 6 mg/kg bw for the 20-nm nanosilver particles and greatly diminished at 6 mg/kg bw for the 100-nm particle size (only dose level tested). Mitogenic responses of spleen B and T cells to concanavalin A remained unaffected. In the blood, the mean corpuscular volume and mean corpuscular hemoglobin of erythrocytes were decreased as were relative lymphocyte numbers (from 90 to 78 %), while IgM, IgE, absolute and relative granulocyte numbers were increased. Overall, the intravenous study indicated that nanosilver may have an immunotoxic potential.

Mary Boudreau (Federal Drug Administration, USA) summarized results from studies of the US National Toxicology Program (NTP) on well-characterized silver nanoparticles of discrete sizes in rats. A toxicokinetic study investigated citrate-stabilized particles of 10, 75 and 110 nm and of silver acetate. Administration was oral (10 mg/kg) or intravenous (3 mg/kg), and silver mass was determined in blood by ICP-MS. In the intravenous study, blood silver concentrations decreased rapidly for nanoscaled silver and less so for the salt. This was followed by re-appearance of silver in the circulation and a subsequent plateau with similar blood silver content for all tested forms on days 2 and 3. In the oral study, *t*_max_ (12 h), *t*_1/2_ (4–5 h) and elimination time (24 h) were similar among silver particles. The area under the curve (AUC) and *c*_max_ increased with decreasing particle size, but did not reach the levels observed with silver acetate. The ADME study investigated the fate of a single oral administration of 20 mg/kg and discovered minimal absorption from this regimen with 0.002–0.006 % of the dose absorbed for nanosilver and approximately 0.016 % for silver acetate at a recovery of 90–120 %. Traces accumulated in the blood, excretory organs and urine. The major route of elimination was via feces within 48 h. In the 90-day subchronic study, 9, 18 or 36 mg/kg of 10-, 75- or 110-nm silver particles with properties comparable to the particles evaluated in the ADME study, or 100, 200 or 400 mg/kg of silver acetate were administered by gavage. Characterization of all materials by transmission electron microscopy (TEM), dynamic light scattering (DLS) and ICP-MS confirmed test article and dose certifications deviating less ±10 % of target values. One preliminary finding was a reduced weight gain and an increase in morbidity in rats with silver acetate doses ≥200 mg/kg, but not in animals treated with nanosilver particles. Gastroenteritis was identified as the cause of death from silver acetate. Renal disease was noted in the case of three rats treated with nanoparticles but a dose-response relationship was not yet established. Further analysis including effects on gut microflora, gross pathology and histopathology, clinical chemistry and hematology as well as TEM of selected tissues was not completed.

In a further presentation, Saber Hussain (Air Force Research Laboratory, USA) presented data on the effects of size, shape, surface coating and charge on silver particle toxicity. He pointed out that a comprehensive mechanistic understanding of how physical parameters of nanosilver particles are linked to biological effects is still lacking. In vitro observations on rat hepatocytes, alveolar macrophages and keratinocytes suggested that silver nanomaterials exhibit size-dependent toxicity (primary particle size and agglomerate size) and elevated reactive oxygen species (ROS) generation (Carlson et al. 2008; Hussain et al. 2005). Interestingly, there was no linear size-response relationship. Among primary particle sizes of 20, 40 and 80 nm, the agglomerates from medium-sized primary particles showed the highest activity. A comparison of 4- and 20-μm silver nanowires in a human alveolar lung co-culture model did not provide evidence for a major influence on epithelial cell viability—possibly due to effective particle scavenging by co-cultured macrophages—but showed alterations in cytokine expression and matrix metalloproteases turnover. Quantitative differences in IL-6 responses indicated a possible influence of shape on effect size. Furthermore, differences in cytotoxicity toward germline stem cells were demonstrated between hydrocarbon-coated and polysaccharide-coated nanosilver with different sizes between 10 and 30 nm (Braydich-Stolle et al. 2010). It was also noted, however, that over time the coating may degrade in the cellular environment, as analyzed by X-ray photoelectron spectroscopy (XPS). Finally, interference of silver and nanosilver with the epidermal growth factor (EGF) signaling pathway was proposed as the underlying mechanism for ROS generation and, ultimately, loss of cell viability. Silver nanoparticles were observed to reduce EGF-dependent phosphorylation of Akt (protein kinase B) and Erk (extracellular regulated kinase), regulators of cell metabolism, survival and proliferation.

## The extensive application of nanosilver might contribute to the spread of silver resistance mechanisms

### Silver resistance mechanisms

In this session, it was initially discussed whether the putative spread of silver resistance mechanisms, due to the extensive application of nanosilver, may be of major concern. Silver is an effective, broad-spectrum antimicrobial agent commonly used in many medical applications, for instance, as topical antimicrobial agent in bandages for burn wounds, trauma and diabetic wounds or tonsillitis but also in silver-coated catheters and medical devices, dental silver amalgams and was historically used as an agent for treatment of syphilis (e.g., silver arsphenamine). In addition to these essential medical benefits, there is an increasing application of nanosilver in consumer products as coating of clothing (e.g., sports fabrics, sleeping bags, socks), in household products and electric devices (refrigerators, boards, PC boards), as an agent to disinfect water, as growth promoter in agriculture or as coating of food packaging materials.

A brief introduction recalled the mode of action of silver toxicity in microorganisms. Despite its use as antimicrobial agent over decades, our current understanding on the mechanism of silver toxicity in microorganisms is still limited. The main cytotoxic effect results from interaction of silver ions with thiol (sulfhydryl) groups and other functional groups in enzymes and proteins, disrupting essential cellular processes such as respiration and the establishment of proton motive force (Jung et al. 2008). Silver treatment also results in the release of potassium ions from bacterial cells, suggesting that the cytoplasmic membrane is compromised upon silver exposure (Schreurs and Rosenberg 1982). An additional impact might come from its interactions with nucleic acids, though the contribution of this target molecule to toxicity remains ambiguous unclear (Yakabe et al. 1980).

Silver resistance has been reported as early as the 1970s in various clinical relevant species including *Escherichia coli*, *Salmonella* *typhimurium*, *Enterobacter cloacae*, *Pseudomonas stutzeri* and *Acinetobacter baumannii* (McHugh et al. 1975; Annear et al. 1976; Bridges et al. 1979; Hendry and Stewart 1979; Haefeli et al. 1984; Kaur et al. 1985; Deshpande and Chopade 1994). Resistance mechanisms include efflux mechanisms (e.g., encoded by *sil* genes) and extracellular or periplasmic silver binding peptides (*silE*). In addition, mechanisms capable to reduce silver ions into less reactive metallic particles which are subsequently deposited periplasmatically are also assumed to contribute to silver resistance, as described mainly for *P. stutzeri* (Klaus et al. 1999). The resistance determinants may be located on chromosomes and on genetic transmissible elements. The as yet best known resistance mechanism is encoded on a 180-kb IncH1 plasmid called pMG101 (Silver 2003). This conjugative plasmid also confers resistance to mercury, tellurite and several antibiotics and is transferable to a wide spectrum of host bacteria (Gupta et al. 1998, 1999, 2001). It is likely that more resistance mechanisms will emerge in the future as more and more bacteria genomes are getting sequenced and characterized.

### Prevalence of silver resistance

The good news from the conference was that at present prevalence of silver resistance is low (Ip et al. 2006; Jakobsen et al. 2011). Yet, although resistance rates among clinical isolates have remained low despite long-term use, silver resistance determinants may be located on mobile genetic elements that confer resistance to multiple other antibiotics. Lotte Jakobsen (Statens Serum Institut, Copenhagen, Denmark) described the IncHI2 plasmid from an extraintestinal pathogenic *E. coli* isolate which conferred resistance to sulfonamides, streptomycin, gentamicin, tetracycline, potassium tellurite, copper sulfate and benzalkonium chloride besides silver nitrate (Johnson et al. 2006). Another cited study reported a major nosocomial outbreak associated with the multi-resistant highly pathogenic *E. coli* ST131 lineage with evidence of multiple cases of transfer of a multi-resistant silver resistant plasmid to patients’ intestinal flora (Sandegren et al. 2012). The finding of co-resistance demonstrates that selection of silver resistance by other antimicrobials is likely. A further case of concern, which was addressed, lies in the development of cross-resistance (Li et al. 1997). Of particular concern is the cross-resistance toward carbapenems which are now the antibiotics of choice to treat one of the fastest growing resistance problems with both community and hospital acquired infections emerging worldwide, that is, the extended-spectrum β-lactamase (ESBL) producing *Enterobacteriaceae* (Sütterlin et al. 2012). Taken together, silver resistance may be co-selected by the use of many antimicrobial agents just like silver use may co-select or cross-select for resistance toward critically important antimicrobials for human medicine. This may occur in a clinical setting as well as in animal production where growth promoters (e.g., copper) are intensively used since the ban of antimicrobials (Johnson et al. 2006). Thus, monitoring of resistance (and consumption) in patients and in the environment is needed to avoid future spread of silver resistance.

### Impact on the composition of the human skin microbiome

Dirk Höfer (Hohenstein Institute, Germany) discussed the putative adverse impact on the composition of the human skin microflora due to the extensive application of silver or nanosilver especially as coating of clothing (e.g., sports fabrics, sleeping bags, socks). It is well known that the normal skin microflora acts as a barrier against colonization of potentially pathogenic microorganisms and against overgrowth of already present opportunistic microorganisms (Sullivan et al. 2001). This kind of control of growth of opportunistic microorganisms is termed colonization resistance. Consequently, one might expect that the release of antimicrobial silver or nanosilver from textiles might affect the microbiome on the human skin. During the conference, results from a study on the short- and long-term effects of antimicrobial active clothes on the ecological balance of the healthy human skin microflora were reported. The wear trials with 60 volunteers carrying silver-coated textile samples releasing 1.9 or 2.5 ppm of silver showed no significant impact on their skin microflora (Hoefer and Hammer 2011).

### Impact on the wastewater treatment

Besides the putative spread of resistance mechanisms toward silver and antibiotics, silver and nanosilver in wastewater are highlighted as a concern due to the potential impact on the biological treatment processes and its poorly known elimination efficiency in wastewater treatment plants (WWTP). Of particularly concern is the discharge of nanosilver in the form of individual particles thereby receiving waters and effects on non-target organisms by released silver ions. Knowledge on the occurrence of silver particles in wastewater and WWTP is still scarce. Michael Burkhardt (HSR University of Applied Sciences Rapperswil, Switzerland) presented a study on the influence of nanosilver on the treatment performance of a real WWTP (60,000 inhabitants equivalent) and a pilot scale WWTP (70 inhabitants equivalent), and on how nanosilver is distributed in the system, particularly in the excess sludge and treated effluent waters (Burkhardt et al. 2010; Kaegi et al. 2011). The effect of four different silver additives (AgCl 20–500 nm; nano-AgA 5–50 nm; OECD reference material NM-300 K; nano-AgB 5–50 nm) on the nitrification (ammonia oxidation) in activated sludge has been studied in batch reactors. The concentrations of silver were measured by ICP-OES, particulate silver was investigated using analytical electron microscopy in combination with energy disperse X-ray spectroscopy (SEM-EDX, TEM-EDX), and the speciation was assessed using X-ray absorption spectroscopy (XAS). On real scale, concentrations <20 μg/l silver even under worst-case conditions of industrial silver discharge were determined in the influent of the real WWTP. Silver was shown to be eliminated with an efficiency of 95–99 %, efficiently transformed within less than 30 min to insoluble silver sulfide (Ag_2_S) which was mostly attached to sludge flocs enabling further filtration in the pilot scale and full scale WWTP. Individual nanosilver, not associated with sludge flocs, was observed occasionally only. The elimination of silver revealed very high compared to organic micropollutants omnipresent in wastewater. All XAS measurements from the full scale WWTP, including samples of the influent, effluent and the digested sludge, confirmed that silver exists as Ag_2_S. The investigated nanosilver additives are readily converted into Ag_2_S in the real-life wastewater matrix. Silver concentrations <0.5 μg/l in effluent waters of municipal WWTP are measured. Ag_2_S is nearly insoluble with a benign ecotoxicity profile compared to free silver ions. Effects on the nitrification process (microbial activity) were not detected even after the addition of 1 mg/l silver corresponding to 250 mg silver in dried sludge. Since the vast majority of nanosilver was found: (1) to be attached to the activated sludge flocs, (2) eliminated by more than 95 % with excess sludge and (3) fully transformed to Ag_2_S with apparently no release of silver ions; Burkhardt concluded that there is no need for action to take special measures for nanosilver removal in the area of WWTP. However, it was argued in the subsequent discussion that a number of published results only refer to tests under artificial laboratory conditions (matrix, concentration, coating) which give only limited insight into environmental behavior in real wastewater.

## Nanosilver containing products are or will be regulated by different legislations around the globe

Depending on their actual use and functionality claims, nanosilver as the active substance and/or nanosilver containing products are or will be regulated by different legislations around the globe. Examples are the European Cosmetics Regulation (Regulation (EC) No 1223/2009), the European Biocidal Product Regulation (BPR, Regulation (EU) No 528/2012) or the US Federal Insecticide, Fungicide and Rodenticide Act (FIFRA).

### Assessment and regulation of nanomaterials under the European Biocides Regulation

Isabel Günther (BfR, Germany) provided the details on the new Regulation (EU) No 528/2012, which replaces the older Biocidal Product Directive (BPD, 98/8/EC) from September 2013 on. For the first time in Europe, the BPR explicitly makes provisions not only for labeling, but also for the assessment of products containing nanomaterials. Like the preceding Biocidal Product Directive, the new BPR foresees a two-step procedure for the final authorization of a biocidal product. In the first step leading to approval (previously: Annex I listing), the active substances undergo a rigorous hazard characterization with peer review by all member state competent authorities, and at least one safe use has to be demonstrated for a respective example product. Considering the uncertainty about the risk-related properties of nanomaterials, the new regulation clarifies that any approval for a biocidal active substance will not include its nanoform unless specified, and a separate dossier will usually need to be submitted. Applicability and reliability of the methods used for testing of nanoscaled active substances are to be justified by the applicant, and adaptations to existing test protocols have to be made, if required. In addition, new test methods specific for nanomaterials will have to be developed. It becomes readily apparent that the risk assessment of nanomaterials to be performed under this regulation may become challenging, and for harmonized evaluation specific guidance has to be developed. Any biocidal products authorized in the second step of the assessment according to the BPR will have to be labeled as “nano” next to any nanoscaled ingredient.

### EPA’s approach to nanosilver risk assessment and regulation

Jed Costanza (Environmental Protection Agency, United States; Costanza et al. 2011) illustrated key elements with respect to human health risks of a US EPA decision about the first registration for an explicitly nanosilver pesticide to be used as textile preservative (EPA-HQ-OPP-2009-1012-0064). The material was described as 1–10-nm silver particles with some particles in the 50-nm range sintered onto amorphous silica. The risk assessment of the pesticide considered occupational as well as consumer exposure to the material, as well as silver nanoparticles, and ionic silver released by degradation. It was based on the currently available data, making conservative assumptions to compensate for knowledge gaps. The toxicity information submitted by the applicant included animal data on acute oral, dermal and inhalation toxicity as well as eye irritation with the product. This was complemented by a characterization of the potential occupational exposure from handling nanosilver powder via the inhalation and the dermal route. For consumer exposure, a scenario in which a 3-year-old toddler is exposed daily to a fresh nanosilver-coated jumpsuit was considered worst case and formed the basis for risk characterization. The US EPA found that the nanosilver risk assessment presented unique challenges with regard to not only the experimental database, but also the methodology of assessment, thus prompting the installation of an external advisory panel. The panel concluded that hazard information for ionic and metallic silver cannot be read across to the nanomaterial. Only a minority of the panel supported bridging to silver materials of similar size and essentially identical physicochemistry. On this basis, risk was assessed using the NOAEL from a 90-day rat inhalation study on uncoated nanosilver of 6–55 (mean 18) nm (Sung et al., 2009) and a 28-day mouse oral (presumably gavage) study with silver particles measuring on average 42 nm (Park et al. 2010). Interestingly, the immunological effects reported in the oral study on mice provided an endpoint that was by a factor of 60 more sensitive than an oral 90-day study in rats with 56-nm silver particles (Kim et al. 2010) performed according to OECD test guideline 408. A dermal repeated-dose toxicity study with nanosilver was not available. The US EPA also identified concerns and data gaps for neurotoxicity, developmental and reproductive toxicity, as well as deficiencies in the database on mutagenicity of nanosilver. An additional uncertainty factor of 10 was applied to account for these deficiencies in risk characterization, resulting in a “no risk” statement for consumer as well as for textile manufacturing workers (taking into account appropriate risk mitigation measures).

EPA’s finding that the proposed use of the pesticide would not cause unreasonable adverse effects on the environment that did not require consideration of the benefits from the use of the pesticide because no risk concern was identified. However, EPA did consider some benefits from the use of the pesticide in determining that granting a conditional registration pending development of additional data would be in the public interest. For example, one public interest benefit identified was an up to 200-fold reduction in silver released into the environment due to the replacement of registered conventional forms of silver by the nanosilver material.

This conditional registration requires the manufacturer to close gaps in the data set for the product in a tiered 4-year testing program. Tier 1 includes endpoints such as particle size distribution and surface area, degradation kinetics, leaching and dermal/inhalation/laundry exposure estimation, as well as a 90-day inhalation and dermal toxicity assay, a reproductive/developmental toxicity screening test and an in vitro micronucleus assay. Tier 2 may require similar testing for any nanoscaled silver released from the composite product.

### The Swedish chemicals agency’s (Kemikalieinspektionen KEMI, Sweden) approach to nanosilver risk assessment and regulation

Ulrike Frank pointed out the fact that in consumer goods silver is used with the aim to employ a biocidal effect. This effect can be reached by the release of silver ions from different silver compounds or silver forms in contact with water. The application of nanoscaled silver or nanosilver composites is only one possibility besides the use of silver salts, silver ion exchangers, silver composites and elemental silver.

Silver is a broad-spectrum microbiocide and has the advantage that the technical handling is comparatively easy. Moreover, so far silver has a better reputation than organic biocides. This makes it feasible to add silver to a great variety of consumer goods, either via incorporation into the polymer material, or via a silver containing coating.

The selling point of silver-treated articles is the demand for “hygienic” goods, or the general fear of bacteria. This fear is differently strong in different cultures. The demand for such products is generally higher in the United States and in East Asia than in Europe.

The majority of silver containing biocidal products contain silver in microscale rather than in nanoscale. The aim of nanoscaled silver as well as of microscaled silver is to release silver ions, as the ions cause the biocidal effect. Consumers are in the first place exposed to silver ions. In the environment, silver ions are rapidly complexed with inorganic sulfides and organo-sulfur compounds. Whether consumers and the environment are exposed to nanoscaled silver has to be shown during the evaluation. Generally, the modeling of exposure is very demanding due to the great variety of consumer articles and their different uses.

### Austria’s approach to nanosilver risk assessment and regulation

Alexander Zilberszac (Austrian Federal Ministry of Health, Austria) talked about the Austrian approach to risk governance for nanosilver. Risk governance is based on the process of risk assessment and includes policy-driven risk management as well as communication of risk management decisions. It also involves the dialog with or between stakeholders. Whenever the scientific basis of risk assessment is limited by methodological or data constraints, risk management and communication measures will dominate the governance process. In the case of nanosilver-based consumer products, a study commissioned by the Federal Ministry of Health concluded that “… an extensive use of nanosilver at low concentrations in consumer products might promote the development of allergies and multi-resistant bacteria.” This conclusion was supported by the opinion of the BfR concerning this matter (BfR 2010). Considering that silver and nanosilver have a high value for certain medical applications, notably treatment of burn victims, the situation of scientific uncertainty prompted a policy response. On July 8, 2011, the Austrian Parliament unanimously adopted a resolution requesting the government “to take all necessary measures to ensure the therapeutic efficacy of nanosilver in human medicine.” The government is now following parallel approaches to implement the Parliament resolution, namely enforcement of existing regulation, communication with consumers as well as supporting relevant research activities and voluntary/private initiatives. With regard to law enforcement, the provisions of the Regulation on the Provision of Food Information to Consumers with respect to labeling of technical nanomaterials (Regulation (EU) No 1169/2011) in addition to the Cosmetics Regulation (1223/2009) and the Biocidal Products Regulation (528/2012) were emphasized. The Federal Ministry of Health further took lead in the installation of the nanoinformation platform (NIP), which is intended to provide an information portal for the interested general public to facilitate opinion making. A NANO Environment, Health and Safety Program (NANO EHS) was set up based on the Austrian Nanotechnology Action Plan. It is the strategic objective of this program to fill knowledge gaps that limit the environmental, occupational and consumer risk assessment for applications of nanomaterials including nanosilver. Last but not least, measures have been installed to ensure that administrations purchase goods and services taking into account potential risks from nanosilver. In particular, the city of Vienna must “…make sure to avoid buying cutting boards or packaging materials with antimicrobial nanosilver coatings,” and may “…not allow antimicrobial paint additives (e.g., nanosilver) for routine applications.” Similar provisions apply for nanosilver coatings in textiles and household appliances. It is also intended to include respective text passages in all sets of criteria for the Austrian eco-label “Öko-Kauf Wien.”

## Conclusions

### Identity and analytics

The enormous number of possible variants of silver nanoparticles is the result of the simplicity of silver nanoparticle syntheses. This huge variety of different silver nanoparticle entities may also be the reason for the absence of certified reference materials for silver. Thus, any real generalizations on what sort of behavior would be expected from any particular material are very difficult to make. A careful characterization on a case-by-case basis is needed to avoid errors coming from the apparently different behavior of nanoparticles due to the use of unknown stabilizing agents. Additionally, the knowledge about the dissolution rates of silver is likely to be extremely critical as variations in the dissolution behavior are strongly related to the physico-chemical properties of the examined nanoparticles.

The biggest challenge so far is to gain a better understanding of the reciprocal effects of nanoparticles when in contact with biological media. Single particle ICP-MS has made a promising progress to overcome some of these hurdles and is able to detect inorganic, non-porous nanoparticles which are composed of one material in complex media. With the completion of current ongoing EU projects, the first finalized validation studies combined with standard operation procedures are expected to become available. However, there are still major gaps related to commonly agreed guidelines for sample preparation and central analytical techniques like electron microscopy.

### Human exposure

Silver nanoparticles are released from a growing number of commercially available products. Skin and respiratory tract were identified as relevant routes for exposure. In this context, the barrier function of the skin, in particular of damaged skin, has to be taken into account in any exposure assessment. Likewise, the inhalative route will have to be addressed in more detail in order to model exposure levels. Next to production techniques, multiple usage and product aging were identified as impact factors for nanoparticle release. These observations indicate a need for case-by-case exposure assessments. Further research has to consider aging processes as a basis for scientific establishment of worst-case assumptions and refinements to more realistic exposure levels. Respirable particles may occur due to spraying or abrasion processes. Sanding procedures of macroscopic surface coatings revealed no generation of free engineered nanoparticles (additives or pigments), previous aging lead to coarser particle size distributions irrespective of the original nanoparticle content. Workplace measurement techniques for aerosols and dry powders are already part of tiered approaches to quantify exposure levels. However, consumer exposure scenarios are only starting to be developed and exposure measurement data are still rare.

### Toxicological aspects

Clearly, the availability of valid toxicological data and information on toxicokinetics has been improved in recent years thus reducing the degree of uncertainty in hazard and risk assessment for nanoforms of silver. Comparability of studies requires thorough characterization of test materials as well as further adjustments and developments in testing design, methodology and analysis. The toxic principle of nanosilver, that is, whether toxicity is ion or particle driven, yet has to be elucidated and analysis of the toxicological profile has to be adjusted accordingly. With regard to particles characteristics, it was noted that physical parameters are directly linked to its biological and toxicological effects. Particularly, the size of primary particles and their most prevalent agglomerates, the size distribution, shape and coating may alter the strength of an effect by orders of magnitude. Silver nanoparticles accumulate in liver and spleen and may affect immunogenic factors after having become systemically available. Key aspects to be addressed in the future are the unequivocal detection of nanosilver particles, their absorption, fate and toxic response. This includes addressing issues such as secondary particle formation, “Trojan horse” effects and interaction of silver particles with biological structures. One has to intensify particle toxicology and learn to understand the differences to soluble chemicals toxicology. This is most striking in terms of barrier penetration, unusual exposure routes (e.g., the olfactory bulb) and accumulation in the body. As existing information showed distribution of silver to brain and testis, testing should also address potential neuro- and reproductive toxicity of nanosilver. The appropriate investigations include kinetics, long-term toxicity testing, standardized, harmonized and predictive in vitro models and their correlation with in vivo data.

### Microbiological aspects

Various clinical relevant species use either efflux mechanisms, extracellular or periplasmic silver-binding peptides or mechanisms capable of reducing silver ions into less reactive metallic particles. It is likely that more resistance mechanisms will emerge in the future as more and more bacteria genomes are getting sequenced. It was common sense on the workshop that the use of silver may lead to the selection of silver resistant bacteria. Since conjugative silver resistance plasmids also confer resistance to several antibiotics and are transferable to a wide spectrum of host bacteria, the selection of silver resistant bacteria may coincide with the selection of antibiotic resistant bacteria. Yet, resistance rates among clinical isolates have remained low despite long-term use. A further issue of concern, which was addressed, lies in the development of cross-resistance. Especially worrisome is the cross-resistance toward carbapenems. Thus, monitoring of resistance (and consumption) in patients and in the environment is needed to avoid future spread of silver resistance.

With respect to environmental behavior of silver, it was proposed that there is no need for action to take special measures for nanosilver removal in the area of WWTP, since the vast majority of nanosilver is converted into insoluble Ag_2_S, found to be attached to biological particles and more than 95 % of silver is eliminated by activated sludge during the wastewater treatment process. Based on these results, it was suggested that nanosilver released to wastewater may have negligible ecotoxicological effects in the aquatic environment. However, in the subsequent discussion, it was argued that a number of published results only refer to tests under artificial laboratory conditions which do not reflect the process conditions from real life and, therefore, may present misleading results to relevant mechanisms.

### Risk assessment and regulation of nanosilver

The presentations and discussion on risk assessment and regulation of nanosilver applications showed that different approaches can be assumed in a data deficient situation. Taking a more “precautionary approach” law enforcement and communication measures were chosen in Austria to limit the use of nanosilver while supporting further research in the frame of a Nanotechnology Action Plan. In the United States, registration of a nanosilver textile preservative was granted weighing uncertainties arising from knowledge gaps against benefits, namely potential reductions in the overall use quantity of silver as a biocide, and—at the same time—linking this registration with the implementation of a testing program to be conducted by the manufacturer. In Europe, the regulation of biocides has been enhanced to specifically address definition, assessment and labeling of nanobiocides such as nanosilver in the near future.
